# Advances in Photoacoustic Imaging of Breast Cancer

**DOI:** 10.3390/s25154812

**Published:** 2025-08-05

**Authors:** Yang Wu, Keer Huang, Guoxiong Chen, Li Lin

**Affiliations:** 1 College of Biomedical Engineering and Instrument Science, Zhejiang University, Hangzhou 310027, China; 12315040@zju.edu.cn (Y.W.); 12315039@zju.edu.cn (K.H.); 2 Hangzhou First People’s Hospital, Hangzhou 310006, China

**Keywords:** photoacoustic imaging, breast cancer, early screening, diagnostic accuracy, therapeutic evaluation

## Abstract

Breast cancer is the leading cause of cancer-related mortality among women world-wide, and early screening is critical for improving patient survival. Medical imaging plays a central role in breast cancer screening, diagnosis, and treatment monitoring. However, conventional imaging modalities—including mammography, ultrasound, and magnetic resonance imaging—face limitations such as low diagnostic specificity, relatively slow imaging speed, ionizing radiation exposure, and dependence on exogenous contrast agents. Photoacoustic imaging (PAI), a novel hybrid imaging technique that combines optical contrast with ultrasonic spatial resolution, has shown great promise in addressing these challenges. By revealing anatomical, functional, and molecular features of the breast tumor microenvironment, PAI offers high spatial resolution, rapid imaging, and minimal operator dependence. This review outlines the fundamental principles of PAI and systematically examines recent advances in its application to breast cancer screening, diagnosis, and therapeutic evaluation. Furthermore, we discuss the translational potential of PAI as an emerging breast imaging modality, complementing existing clinical techniques.

## 1. Introduction

In recent years, the incidence of breast cancer has been continuously rising, making it the leading cause of cancer-related mortality among women worldwide [1]. An analysis of data from 185 countries revealed approximately 2.3 million new cases and 670,000 deaths from breast cancer globally in 2022, with projections indicating a 38% increase in cases and a 68% rise in deaths by 2050, posing a serious threat to women’s health [2].

Breast cancer develops through a multistep transformation process, progressing from precancerous lesions to carcinoma in situ and eventually to invasive carcinoma [3]. This process is driven by cumulative genetic and epigenetic alterations that disrupt regulatory pathways. Mutations in tumor suppressor genes (e.g., TP53, BRCA1/2) and activation of oncogenes (e.g., PIK3CA) lead to uncontrolled proliferation and impaired DNA repair [4,5,6,7]. Epigenetic changes, such as promoter hypermethylation and histone modification, silence key genes involved in cell cycle regulation and apoptosis. Carcinogenic factors—such as inherited susceptibility, hormonal stimulation, and environmental exposures—induce irreversible molecular alterations in normal breast epithelial cells, promoting clonal expansion and the formation of atypical hyperplastic lesions. At this stage, signaling pathways like PI3K/AKT/mTOR and HER2/EGFR are frequently activated, supporting cell survival and growth [8]. Continued proliferation increases genomic instability, favoring further mutations and transition to carcinoma in situ. Loss of E-cadherin and disrupted cell junctions facilitate early local invasion. The tumor microenvironment also contributes to progression. Hypoxia activates HIF-1α signaling, inducing VEGF expression and neovascularization [9,10]. These abnormal vessels supply nutrients and provide routes for malignant cells to invade surrounding tissues. Epithelial–mesenchymal transition (EMT), mediated by factors such as TWIST1, SNAIL, and ZEB1, enhances invasiveness and metastatic potential. Eventually, invasive carcinoma develops, with invasive ductal carcinoma (IDC) being the most common subtype, accounting for approximately 80% of all breast cancer cases in women [11,12,13,14].

At the carcinoma in situ stage, cancer cells remain well-defined and clearly separated from normal breast tissues [15,16], with neither lymph node involvement nor distant metastasis, resulting in excellent prognosis and high survival rates. Early screening and accurate diagnosis are crucial for reducing mortality. Furthermore, effective treatment requires precise diagnosis and must consider individual patient differences. Therefore, timely feedback, therapeutic efficacy evaluation, and prognosis assessment are essential throughout the course of treatment. Medical imaging techniques serve as primary or complementary tools in screening, diagnosis, and treatment monitoring.

### 1.1. Conventional Imaging Methods of Breast Cancer

Breast cancer screening is aimed to identify potential precancerous lesions in breast tissue through noninvasive, efficient, and sensitive methods. Currently, conventional clinical screening techniques include clinical breast examination, mammography, ultrasound (US), and magnetic resonance imaging (MRI). Mammography is the most widely used screening technique, seen as the gold standard in western countries. It can detect microcalcifications smaller than 100 μm so that allowing identification of small lesions [17,18] with the advantage of low-dose radiation. However, its sensitivity ranges from 69% to 90% [19,20], especially lower in young women with dense breast tissue. Concerns about breast thickness and radiation safety further restrict its application in some regions and countries. As a supplementary tool to mammography, US is unaffected by breast density and offers advantages such as low cost, high safety and a relatively short examination time of approximately 15 to 30 min [21,22,23,24]. However, its diagnostic performance is highly operator-dependent, with limited sensitivity to small early-stage lesions and low specificity. In addition, high risk population is recommended to breast MRI, allowing for imaging of deep breast lesions and axillary lymph nodes, among other specific areas with the disadvantages of high cost, long imaging time, and the injection of contrast agent.

Since each imaging modality has its own strengths and limitations, breast cancer diagnosis requires a comprehensive evaluation based on clinical presentation, imaging findings, and histopathological analysis. The imaging-based diagnosis of breast tumors typically involves sequential use of ultrasound, mammography, and MRI. By combining the high sensitivity of mammography for detecting microcalcifications with the ability of ultrasound to differentiate between cystic and solid masses, the benign or malignant nature of a lesion can be preliminarily assessed, and its location determined. If both methods fail to yield a conclusive diagnosis, biopsy or MRI is required. Due to the limited specificity of ultrasound and mammography, more than 75% of patients who undergo biopsy are ultimately diagnosed with benign lesions [25], placing a significant burden on both patients and clinicians.

Overall, although conventional imaging methods are widely used in clinical, they still have fundamental limitations. In early breast cancer screening, there is a lack of medical imaging techniques that are fast, safe, standardized, equally effective for dense breasts, and highly sensitive. The low diagnostic specificity of ultrasound and mammography, along with the lengthy imaging time required for MRI—which still cannot achieve true real-time imaging despite recent acceleration techniques such as parallel imaging and compressed sensing that improve its temporal resolution—continues to hinder accurate early detection of breast tumors [26]. Furthermore, no single imaging technique is sufficient for real-time treatment monitoring, dynamic therapeutic evaluation, or accurate prognostic assessment during neoadjuvant chemotherapy (NAC). Existing methods for intraoperative margin assessment are also inadequate. Therefore, there is an urgent clinical need for a novel imaging technique that can complement current methods and more effectively address these critical challenges.

### 1.2. Principles and Advantages of PAI

Photoacoustic imaging (PAI) is an emerging non-invasive biomedical imaging technique based on the photoacoustic (PA) effect. When biological tissues absorb short-pulsed laser light, they undergo thermoelastic expansion, resulting in the generation of ultrasound waves. By detecting and reconstructing acoustic signals, it is possible to obtain images that reflect the optical absorption distribution within the tissue. The generation of the acoustic wave can be described by the following equation [27]:

(1) $$p_{0}=\Gamma \eta_{\mathrm{th}}\mu_{a}F$$

where $p_{0}$ represents the photoacoustic pressure field, Γ is the Grüneisen parameter (dimensionless), $\eta_{\mathrm{th}}$ denotes the percentage of absorbed light converted into heat, $\mu_{a}$ is the optical absorption coefficient (cm^−1^), and F refers to the local optical fluence (J/cm^2^).

Compared with conventional optical imaging, PAI effectively overcomes the resolution degradation caused by tissue scattering, achieving high resolution and contrast at centimeter-scale imaging depths. In addition, PAI offers several unique advantages: it enables multi-wavelength functional imaging to reflect key physiological parameters such as blood oxygenation and metabolism; it can also be integrated with ultrasound, fluorescence, and other modalities for multimodal and cross-scale imaging, applicable across various biological levels from cells to organs. Furthermore, PAI features fast imaging speed, making it suitable for real-time monitoring of dynamic processes such as blood flow and drug metabolism [28,29,30,31].

Based on the above advantages, PAI plays an important role in modern medicine [32] and has been widely applied in the diagnosis of tumors [33], neurological diseases [34], vascular disorders, and arthritis [35]. In tumor imaging, PAI can provide critical physiological parameters such as blood flow characteristics and oxygen saturation (sO_2_) within the tumor microenvironment, thereby compensating for the limitations of traditional imaging techniques in acquiring functional information. This capability is of great significance for evaluating tumor growth, progression, and therapy. The tumor microenvironment is a complex cellular ecosystem influenced by chemical and physical signals that contribute to tumor heterogeneity, invasion, and metastasis [36]. PAI can measure blood oxygen saturation based on the different absorption spectra of oxyhemoglobin (HbO_2_) and deoxyhemoglobin (Hb) [37], thereby reflecting the hypoxic state of the tumor microenvironment. In addition, PAI can use single-wavelength irradiation of biological tissues to reconstruct the distribution of tumor vasculature and monitor angiogenesis. Furthermore, curvature, diameter, and density can also serve as imaging biomarkers for quantitative data analysis [36,38].

This review first outlines the current clinical needs in breast imaging and highlights the translational potential of PAI. It then presents representative studies from various research groups on breast tumor screening, diagnosis, and treatment monitoring. Key aspects such as system design, highlighted clinical studies, and major findings are analyzed, accompanied by objective evaluations. Finally, we discuss the future prospects and challenges of PAI in clinical breast applications.

## 2. Breast PAI Systems

PAI systems can be divided into three primary categories: photoacoustic microscopy (PAM) [39], photoacoustic endoscopy (PAE) [40], and photoacoustic computed tomography (PACT) [41]. PAM is mainly employed in applications such as skin imaging and tumor margin detection, and it complements PACT in terms of imaging scale [42,43,44]. PAE systems are typically developed by miniaturizing either PACT or PAM systems and are suitable for imaging intracavitary tissues and nearby organs such as the rectum and prostate [45,46]. Both PAM and PAE achieve micrometer-level spatial resolution at millimeter-level imaging depths [27]. Compared with PAM and PAE, PACT offers centimeter-scale imaging depth and wide-field rapid scanning. However, its spatial resolution is generally lower, around several hundred micrometers. PACT has been widely applied in imaging whole small animals and human organs such as the breast [47,48,49].

Breast PAI systems are mainly classified into two categories: bed-based systems designed for whole-breast scanning, and handheld systems adapted from ultrasound probes for localized lesion imaging [50]. Furthermore, other novel PAI systems are also introduced. In Table 1, we summarize the photoacoustic imaging systems.

### 2.1. Bed-Based Imaging PAI Systems

**Figure 1:**
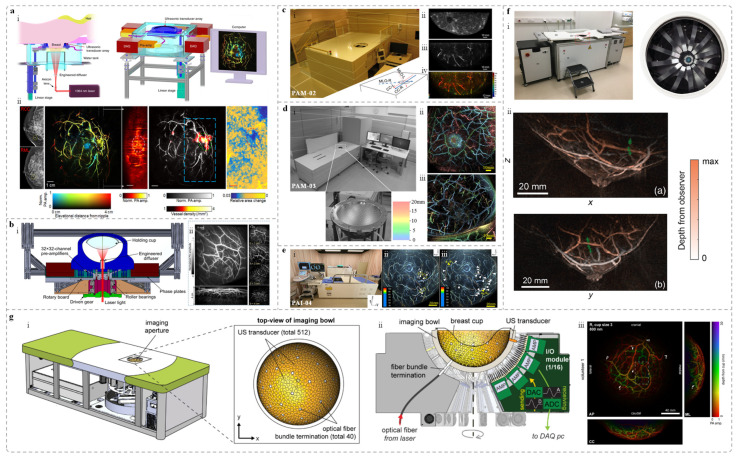
Bed-based whole-breast PAI systems and representative images. (**a**) The SBH-PACT system and imaging results [48]: (i) System cutaway view (left) and perspective view (right); (ii) From left to right: mammogram, depth-encoded PA image, sagittal PA image, PA image with vascular density overlay, and photoacoustic elastography image. (**b**) Bed-based 3D-PACT system [51]: (i) System cross-section view after removing the imaging platform; (ii) 3D PA breast images of a subject [from left to right, vascular projection image of the breast (top) and vascular projection image of the breast lateral view (bottom), and cross-sectional image at different coronal planes from the nipple to the chest wall. Each cross-sectional image represents a projection of a 1 cm-thick slice of the breast.]. (**c**) PAM-02 system and imaging results [54]: (i) Photograph of the system; (ii) US image from the system; (iii) PA structural image; (iv) PA functional image. (**d**) PAM-03 system and representative images [55]: (i) Photograph of the system and transducer array layout; (ii) and (iii) Imaging results from two healthy volunteers. Both of them are coronal views and colored the signals according to the depth using the color chart. (**e**) PAI-04 system and PA images [56]: (i) Photograph of the system; (ii) and (iii) PA images of breasts from two healthy participants. Waite rows mean the measured positions for the reproducibility evaluation of the S-factor in the breast. The measured ranges of the S-factor are indicated by the yellow dotted lines. Through measurements, it is possible to distinguish between arteries and veins in the breast. (**f**) PAM 2 system and imaging results [57]: (i) Photo of the system (left) and imaging tank (right); (ii) Color local maximum intensity projections (LMIPs) of the left breasts from two healthy volunteers acquired at 755 nm, showing sagittal (left, max depth 110 mm) and transverse (right, max depth 100 mm) views. Green crosses follow a blood vessel at a depth of 22 mm from the skin surface. (**g**) PAM3 breast imaging system and imaging results [58]: (i) Schematic of the PA-US imaging system and the top-view of imaging bowl. (ii) Cross-sectional view of the system core. (iii) Full-SOS-compensated maximum intensity projection (MIP) images in the anterior–posterior, medial–lateral, and cranial–caudal views of the right breast of a healthy volunteer.

The single-breath-hold photoacoustic computed tomography (SBH-PACT) system was developed by Lin et al. [48]. This system integrates a non-focused 512-channel full-ring ultrasound array (diameter: 220 mm; center frequency: 2.25 MHz; bandwidth: 95%) combined with laser illumination at a wavelength of 1064 nm (pulse repetition rate: 10 Hz; pulse width: 8–12 ns). In addition, a 750 nm light source is utilized to generate sO_2_ maps. During the imaging, the patient lies in a prone position, with the breast gently compressed and positioned within the imaging window. A linear stage drives the transducer array to complete a full-breast scan within ~15 s, which can be accomplished in a single breath-hold. The system achieves an in-plane resolution of 0.26 mm, an axial resolution of 5.6 mm, and an imaging depth of up to 4 cm in vivo. As shown in Figure 1a, SBH-PACT provides high-quality vascular imaging and facilitates the detection of occult tumors within complex vascular networks.

In addition, Lin et al. also developed a three-dimensional photoacoustic computed tomography (3D-PACT) system based on a hemispherical housing embedded four arc-shaped ultrasound arrays. Each array has 256 elements and the center frequency is 2.25 MHz [51]. The 3D-PACT achieves isotropic resolution of 370 μm and the imaging depth of 4 cm in vivo of human breast. During breast imaging, the healthy volunteer lies prone on the imaging platform, with the breast gently compressed against the chest wall using a molded bowl. A 1064 nm laser illuminates the breast from below, while the four arc-shaped arrays rotate 90° around the laser axis to acquire a dense hemispherical sampling matrix. Volumetric imaging of the breast is completed within 10 s, clearly visualizing vascular structures from the skin surface to the chest wall (Figure 1b).

Kyoto University and Canon Inc. have jointly developed three prototypes of PAI systems, referred to as PAM-01, PAM-02, and PAM-03 [52]. Both PAM-01 [53] and PAM-02 [54] utilized a planar-scanning detector with bi-directional light delivery from both the head and tail ends of the probe. PAM-01 employs four-wavelength laser illumination (756, 797, 825, and 1064 nm) and a custom planar ultrasound array consisting of 345 elements, with a center frequency of 1 MHz and 80% bandwidth. The imaging area covers 30 mm × 46 mm per scan, with full-breast coverage achieved through mechanical scanning. Building upon PAM-01, PAM-02 features a new custom planar ultrasound array with 600 elements and a center frequency of 2 MHz (130% bandwidth), which improves spatial resolution from 2 mm to 1 mm. In addition, it incorporates an ultrasound array for US imaging and switches to dual-wavelength illumination. Using PAM-01, the research team imaged 39 breast cancer patients and extracted photoacoustic features related to angiogenesis and localized hypoxia, which were then compared with conventional imaging and histopathological findings. With PAM-02, they imaged one patient with invasive breast carcinoma of no special type (IBC-NST), revealing a centripetal vascular distribution around the tumor and sparse internal spot-like signals (Figure 1c).

The third-generation prototype, PAM-03, employs a hemispherical detector array (HDA) to receive photoacoustic (PA) signals, aiming to overcome the field-of-view (FOV) limitations of previous systems and enhance the visualization of 3D vascular architecture (Figure 1d) [55]. In healthy breast imaging, PAM-03 revealed more detailed vascular characteristics than MRI, demonstrating richer vascular branching and finer morphological features. Subsequently, a clinical study was conducted involving 22 patients with malignant breast tumors. PAM-03 revealed distinct peritumoral vascular abnormalities, including PA signal enhancement, vessel narrowing or discontinuity, and detectable intratumoral signals. In addition, the system demonstrated the ability to monitor treatment-induced changes in vascular morphology and function. These findings underscore the potential of PAI as a noninvasive tool for vascular analysis in tumor diagnosis and therapeutic evaluation. In 2018, the group further introduced the PAI-04 breast PAI system (Figure 1e) [56], which integrated several key optimizations while retaining the external design of the PAI-03. First, the original 2 MHz hemispherical detector array (HDA) was upgraded to 4 MHz, effectively improving the spatial resolution from 0.5 mm to 0.27 mm, as demonstrated in phantom experiments. Second, the laser control was modified from sequential illumination (756 nm and 797 nm) to interleaved pulse-by-pulse switching, significantly enhancing the efficiency of multi-wavelength imaging. Third, a 256-element linear ultrasound transducer array was added to acquire ultrasound (US) images, which were spatially co-registered with the PA images to enable fused PA-US image. This system allows for 3D visualization of tumor-associated vasculature and hemoglobin oxygen saturation, providing additional functional and molecular-level information for breast cancer diagnosis. It is worth noting that PAI-03 and PAM-03 refer to the same system, as confirmed by both literature sources [55,59], along with device photographs in their supplementary materials.

Schoustra et al. developed a breast PAI system, referred to as PAM 2 [57], which utilizes dual-wavelength lasers at 755 nm and 1064 nm and can operate in either single-wavelength or dual-wavelength modes (Figure 1f). The primary laser beam is split into two paths using a beam splitter, enabling illumination from both the bottom and the side of the breast. The energy distribution between the two paths can be adjusted by using different types of beam splitters. Two illumination modes were evaluated in the study: a 67/33 mode, in which 67% of the energy is delivered from the bottom and 33% via fiber bundles from the side; and a 1:1 mode, where the energy is equally split between bottom and side illumination. PAM 2 is equipped with 12 arc-shaped ultrasound arrays, each consisting of 32 elements with a center frequency of 1 MHz. The detector array performs rotational scanning to acquire multi-angle images. Imaging results from two healthy female volunteers demonstrated clear visualization of breast contours, nipple anatomy, and internal vasculature. Figure 1f shows the local maximum intensity projections (LMIPs) of the left breast from one volunteer. LMIP is a volume reconstruction method that displays the local maxima of voxel intensities [60]. In addition, using PAM 2, the team imaged 30 breast cancer patients, 19 of whom had malignant tumors [61]. The system provided clearly delineated vascular structures for all subjects. Notably, the PA images of one patient showed multiple high-intensity spots located posterior to the nipple, which spatially corresponded to the tumor region identified by physical examination and mammography.

The PAM3 [58], a photoacoustic-ultrasound (PA-US) breast imaging system developed by Dantuma et al., is mounted on a supporting frame equipped with a 2.5 m × 1 m patient bed. The system enables three-dimensional, multi-wavelength PAI and incorporates volumetric speed-of-sound (SOS) correction to reduce wavefront in PA image reconstruction of heterogeneous breast tissue, thereby improving imaging depth and accuracy. During scanning, the volunteer lies prone with one breast positioned through an opening into a 26 cm-diameter hemispherical bowl. The bowl contains watertight inserts housing 40 optical fiber bundles, 512 ultrasound transducers (1 MHz), and 2 thermometers. The excitation source comprises two identical units, each consisting of a Q-switched Nd:YAG pump laser, a DKDP crystal-based second harmonic generator, and a BBO crystal optical parametric oscillator (OPO). The two pump lasers are triggered with a 90 ns delay to avoid temporal overlap, and their output beams are combined into a single beam via a Glan–Taylor polarizer. The bowl rotates 360° to acquire multi-angle data. Each acquisition module controls 32 transducers, and the system software allows customization of parameters such as wavelength, averaging, and rotation step size. They imaged four healthy volunteers. Figure 1g shows depth-encoded images of the right breast of one volunteer at 800 nm after SOS correction, with anterior–posterior, medio–lateral, and cranial–caudal photoacoustic maximum intensity projections (MIPs) clearly revealing complex vascular structures.

### 2.2. Handheld PAI Systems

**Figure 2:**
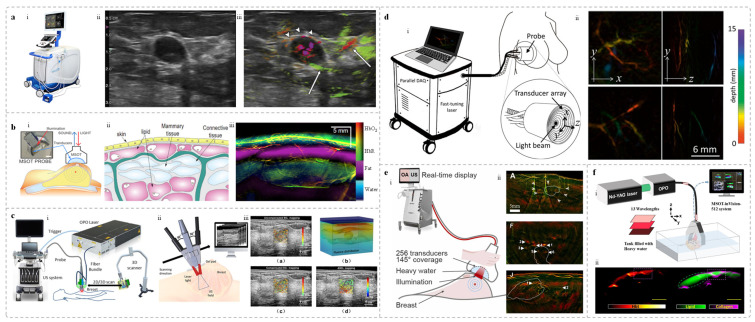
Handheld PAI systems based on localized detection and corresponding breast imaging results. (**a**) Imagio^®^ system and imaging results [64]: (i) Photograph of the Imagio^®^ system; (ii) and (iii) respectively show the US image (left) and the fused PA-US image (right) of a patient with triple-negative invasive ductal carcinoma. (**b**) The MSOT system schematic and imaging results [65]: (i) System diagram and probe photo; (ii) Schematic of breast tissue structure; (iii) MOST imaging of a healthy breast, revealing the layered structure of the breast. (**c**) Handheld device developed from a clinical ultrasound platform and imaging results [66]: (i) US/PA dual-modality system schematic; (ii) Handheld probe illustration; (iii) PA imaging results from a patient with a breast tumor; (**d**) The 3D multispectral PAI system and imaging results [67]: (i) Imaging and probe schematic (enlarged view); (ii) Maximum intensity projections of the breast from a healthy volunteer along the z and x directions with depth-coded color. (**e**) The Acuity Echo^®^ and PA images [68]: (i) System diagram during acquisition; (ii) PA images of three breast cancer patients, with white contours indicating tumor regions. (**f**) Schematic of handheld US-PA probe and functional results [69]: (i) System diagram; (ii) PA image (left) showing total hemoglobin (HbT) distribution and fused image (right) displaying lipid (green) and collagen (magenta); blue represents veins with low sO_2_, red indicates arteries with normal saturation.

The Imagio^®^ breast imaging system (Figure 2a), developed by Seno Medical, is an integrated dual-modality breast imaging platform. It is the first PAI system approved by the U.S. Food and Drug Administration (FDA) [62], and is suitable for the auxiliary evaluation of BI-RADS 3, 4, and 5 breast lesions. The system employs a dual-wavelength laser source at 1064 nm and 757 nm. To reduce artifacts caused by tissue motion during imaging, a delay of approximately 5 ms is introduced between the two wavelength switches. The laser light is delivered through a fiber bundle composed of 200 μm-diameter fibers, with a total bundle diameter of 8.3 mm, and is evenly distributed across two 40 mm × 6 mm optical windows. The optical module incorporates custom-designed lenses and light diffusers to optimize illumination uniformity. The probe consists of a 128-element linear ultrasound array with a bandwidth range of 0.1–12 MHz, and fiber bundles integrated on both sides [63,64]. Tumors ranging from 3 mm to 20 mm in diameter, along with associated vasculature, can be visualized in vivo at depths of up to 40 mm. In addition, the Imagio^®^ system enables analysis of the spatial distribution of oxyhemoglobin (HbO_2_), deoxyhemoglobin (Hb), and total hemoglobin (HbT) in breast tissue.

Diot et al. developed a multispectral optoacoustic tomography (MSOT) system for clinical breast cancer detection (Figure 2b) [65]. The system comprises a 174° arc-shaped ultrasound array with a 60 mm radius, and laser source operating across wavelengths from 680 to 980 nm. Imaging was performed at 28 wavelengths ranging from 700 to 970 nm, utilizing single-frame pulse projection to enable rapid scanning within 0.56 s. The clinical study involving 3 healthy volunteers and 10 breast cancer patients demonstrated the system’s capability to HbO_2_, Hb, total blood volume (TBV), water, and lipids (Figure 2b). The results revealed significant differences in vascular density, structural heterogeneity, and TBV between tumor and healthy tissues. Compared to conventional optical methods, MSOT demonstrated superior tumor detection accuracy and imaging depth, highlighting its strong potential for clinical application.

Li’s team, in collaboration with Mindray, developed a handheld PAI device for breast applications [50,66]. The system integrates a clinical ultrasound platform with a 192-element linear array, operating at a center frequency of 5.8 MHz, and provides FOV of 4.4 cm × 5 cm. An OPO laser with a pulse repetition frequency of 10 Hz is incorporated as the excitation source. The laser beam is delivered through a bifurcated fiber bundle mounted on both sides of the probe at a 30° incident angle (Figure 2c). During imaging, two wavelengths (750 nm and 830 nm) were employed to measure sO_2_. The team imaged 2 breast tumor patients to validate the accuracy of measured sO_2_ in vivo.

Dean-Ben et al. developed a 3D multispectral PAI system [67,70]. The system comprises three main components: (1) A multi-element semi-spherical matrix array with 256 elements (radius: 40 mm; angular coverage: 90°), a center frequency of 4 MHz, and 100% bandwidth; (2) An OPO laser with a pulse duration of <10 ns, a wavelength range of 690–900 nm, an energy density of <15 mJ/cm^2^, and optical power < 200 mW/cm^2^; (3) A data acquisition system with a sampling rate of 40 MHz. The system achieves a spatial resolution of approximately 0.2 mm. In dense breast tissue, it enables high-contrast 3D vascular imaging at depths greater than 2 cm, offering a promising solution for early breast cancer screening (Figure 2d).

In addition to the Imagio^®^ Breast Imaging System, the Acuity Echo^®^ system developed by iThera Medical GmbH has been CE-Marked [68]. The system features a 145° curved transducer array with 256 elements. Compared to linear arrays, the curved design offers a wider FOV and improved image quality. Equipped with a fast-tunable laser, the Acuity system can acquire 28 multispectral images within 1.1 s, thereby reducing motion artifacts and enhancing spectral resolution. Image reconstruction is performed using a filtered back-projection algorithm, which enables real-time imaging and also supports offline reconstruction for further image quality enhancement. The system achieves axial and lateral resolutions of 320 μm and 510 μm, respectively [68]. Various versions of the Acuity system have been successfully applied in clinical studies [71,72,73,74,75,76,77,78,79]. In breast imaging applications, Kukačka et al. utilized the Acuity system and developed advanced data analysis methods to improve the diagnostic accuracy of PAI in breast cancer detection [80]. The PA images revealed irregular structures surrounding the tumors, along with multiple regions of signal enhancement (Figure 2e). Furthermore, the presence of tubular structures within tumor cores indicated abnormal angiogenesis and malignancy-associated vascular distribution.

A customized 2D PA probe was integrated with the MSOT inVision 512-ECHO system (iThera Medical GmbH, Munich, Germany) to perform dual-modality PA/US imaging (Figure 2f) [69]. The probe features a curved array with 256 elements distributed over a spherical surface (curvature radius: 40 mm) covering a 125° angle and working at a center frequency of 5 MHz. Compared to linear arrays, this geometry provides a wider FOV and enhances PA image quality. The study evaluated the feasibility of using the handheld probe to analyze ex vivo breast specimens obtained from patients treated with breast-conserving surgery (BCS). Results indicated that combining PA images with biochemical information (collagen and lipid content) and ultrasound images effectively differentiates malignant from normal breast tissues, aiding margin evaluation in BCS.

### 2.3. Other Forms of PAI Systems

In addition to the table-based and handheld breast PAI systems, several structurally innovative breast PAI devices have also been developed. Zhang et al. introduced a prototype whole-breast imaging system termed photoacoustic synthetic matrix array tomography (PA-Smart), which employs a scanning approach similar to X-ray mammography [50]. During testing, the patient stands in front of the system and positions one breast on a transparent glass plate, which is gently compressed by the bottom of an overlying water tank. This compression reduces breast thickness, thereby improving light penetration and minimizing respiratory motion artifacts. A one-dimensional unfocused linear ultrasound array, housed within the water tank, is driven by an electrically controlled translational stage at a speed of 3 mm/s. The tank bottom is composed of low-density polyethylene (LDPE), whose acoustic impedance closely matches that of breast tissue, facilitating efficient acoustic coupling. The system achieves a spatial resolution of approximately 1 mm, and phantom studies have demonstrated its capability to image a volume exceeding 10 cm × 10 cm × 4 cm within 33 s. However, its clinical applicability remains to be validated, as no in vivo studies have been reported to date.

Another system with a mammography-inspired design is the Dual Scan Mammoscope (DSM), developed by Nyayapathi et al. [81]. The system comprises two opposing water tanks with flexible plastic-film windows that gently compress the breast from above and below, enhancing both patient comfort and optical illumination. Dual 128-element linear ultrasound arrays and co-planar fiber bundles are employed to enable simultaneous top- and bottom-side imaging along the craniocaudal plane. This configuration provides a large imaging volume (up to 7 cm in depth) with approximately 1 mm isotropic resolution. The system supports fast scanning (within 1 min) and operates at laser energy levels within established safety limits. Interleaved photoacoustic and plane-wave ultrasound imaging is achieved using custom software and synchronized data acquisition, offering a radiation-free and portable solution for breast cancer screening, particularly in patients with dense breast tissue. In a clinical study involving 38 patients with various tumor types [82], the DSM system demonstrated its ability to differentiate tumor-bearing from healthy breasts. Tumor regions exhibited larger-caliber vasculature and greater background signal heterogeneity. Preliminary findings also suggested potential for tumor-type classification. These results support the utility of integrated photoacoustic and ultrasound imaging for improved breast cancer detection.

To address system limitations, the team subsequently introduced DSM-2, an upgraded version of the DSM with enhanced mechanical precision and imaging performance [83]. Compared to the original system, DSM-2 features a more stable and precisely machined structure, which reduces transducer misalignment and enables standardized image reconstruction. A laterally shifted round-trip scanning scheme was implemented, doubling the lateral imaging coverage from 85.6 mm to 171.2 mm—sufficient for full-breast imaging without increasing scan time and compromising resolution. Both phantom and human subject studies confirmed that DSM-2 significantly improved lateral coverage while maintaining imaging speed and spatial resolution.

**Table 1:** Summary table of breast PAI systems.

System	Structure	Detector	Laser	Resolution	Imaging Depth	Scan Time	Advantages	Limitations
SBH-PACT (Lin et al.) [48]	bed-based	512-ring array (2.25 MHz)	1064 nm/10 Hz/8–12 ns	In-plane: 0.26 mm; elevation: 5.6 mm	4 cm	~15 s (single-breath)	fast scan; clear vessels	poor elevational resolution
3D-PACT (Lin et al.) [51]	bed-based	4 × 256 arc arrays (2.25 MHz)	1064 nm/10 Hz/8–12 ns	370 μm	4 cm	~10 s	isotropic resolution; fast; clear image	single-wavelength
PAM-02 (Kyoto & Canon) [54]	bed-based	600-planar (2 MHz) + 128-linear (6 MHz)	756 & 797 nm/10 Hz/7 ns	~1 mm	Phantom: ≥45 mm; Breast: ≥25 mm	depends on scan area	better resolution; PA/US fusion	low imaging depth
PAM-03/PAI-03 (Kyoto & Canon) [55]	bed-based	512-HDA (2 MHz)	755 & 795 nm	~0.5 mm	~30 mm	~4 min	shows small vessels missed by MRI	inaccurate sO_2_
PAI-04 (Kyoto & Canon) [56]	bed-based	500-HDA (4 MHz) + 256-linear US	756 & 797 nm/10 Hz	~0.27 mm	<10 mm	~2 min	improved resolution; PA/US + sO_2_ analysis	complex; clinical adaptability unverified
PAM 2 (Schoustra et al.) [57]	bed-based	12 × 32 arc arrays (1 MHz)	755 nm (60 ns) + 1064 nm (5 ns)/10–20 Hz	~1 mm	22 mm	~4 min	adjustable lighting	few elements
PAM3 (Dantuma et al.) [58]	bed-based	512-bowl array (1 MHz, d: 26 cm)	720–860 nm/10 Hz/4.2 ns	axial: ~426 µm	48 mm	~5 min	sound velocity correction; multi-λ imaging	large size; high cost
Imagio^®^ (Seno Medical) [62]	handheld	128 linear (0.1–12 MHz)	757 nm (50 ns) + 1064 nm (15 ns)/5 Hz	axial: 0.42–0.47 mm; lateral: 0.73–0.81 mm	≤40 mm	–	FDA-approved; HbO_2_/Hb quant; US fusion	limited FOV
MSOT (Diot et al.) [65]	handheld	256-curved (5 MHz, d: 12 cm)	680–980 nm (28 λ); 50 Hz/8 ns	200–300 µm	≤30 mm	2–4 min (0.56 s/frame)	High res; vascular mapping	needs photon correction; motion artifacts
Handheld PAI (Li et al. & Mindray) [66]	handheld	192-linear US (5.8 MHz)	750/830 nm/10 Hz	–	–	~40 s (4 cm/0.1 cm/s)	PA/US fusion; real-time; sO_2_ mapping	low frame rate; motion artifacts
3D multispectral PAI (Dean-Ben et al.) [67]	handheld	256-spherical (4 MHz, r: 40 mm)	690–900 nm/<10 ns	~200 µm	≥2.2 cm	–	fast 3D + functional mapping	shallow in dense tissue; motion-sensitive
Acuity Echo^®^ (iThera Medical GmbH) [68]	handheld	256-arc (4 MHz)/256–384 hemispherical cup (8 MHz)	660–1300 nm/≤25 Hz/28 λ in 1.1 s	80–400 µm	4cm (2D detector)	–	fast multi-λ imaging; CE-Marked	cannot assess depth; tissue limits unclear
MSOT inVision 512-ECHO (iThera Medical GmbH) [69]	handheld	256-spherical (5 MHz, 125°)	660–1300 nm/10 Hz	In-plane: 150 µm	5mm	–	high resolution	low imaging depth
PA-Smart (Zhang et al.) [50]	scanning (like mammography)	1D linear (2 × 48)	1064 nm/10 Hz/9 ns	lateral: 1.12–1.57 mm; elevation: 0.60–0.63 mm	4 cm	~33 s	large FOV (10 × 10 × 4 cm^3^)	limited small vessel resolution
DSM (Nyayapathi et al.) [81]	scanning (like mammography)	2 × 128-linear array (2.25 MHz)	1064 nm/6–10 Hz/10 ns	~1 mm; elevation: 1.47 mm (2D), 1.05 mm (3D)	Up to 7 cm	~60 s	unprecedented 7 cm imaging depth	low resolution

## 3. Noninvasive Screening and Diagnostic Capacities of PAI

PAI combines the high optical contrast of optical imaging with the deep tissue penetration of ultrasound, demonstrating strong clinical potential for the noninvasive screening and diagnosis of breast tumors. It provides high-resolution structural and functional information related to tumor-associated angiogenesis, offering additional evidence for the detection and characterization of breast tumors, as well as for determining the necessity of biopsy [84,85]. Furthermore, the quantitative analysis of multiple PA image features—when combined with US image features—facilitates the development of predictive models to assess the probability of malignancy in breast lesions, thereby supporting clinical decision-making [86]. Recent advances in open-source datasets and machine learning algorithms have contributed to higher spatial resolution, reduced artifacts, and improved diagnostic accuracy.

**Figure 3:**
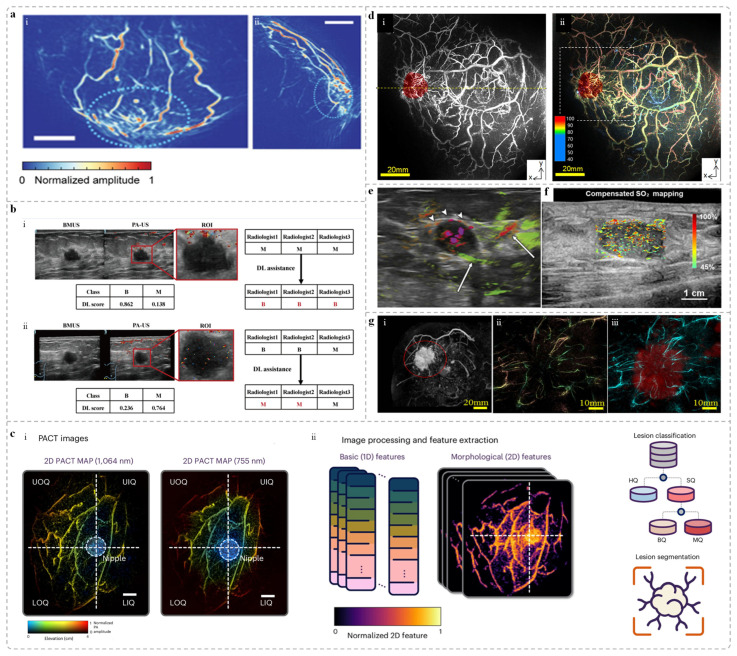
PAI performance in noninvasive breast cancer diagnosis across multiple studies. (**a**) (i) and (ii) Photoacoustic (PA) images acquired using the PAM 2 system, showing transverse and sagittal views of a patient diagnosed with mucinous carcinoma [61] (the dotted line area is the tumor location). (**b**) Typical diagnostic cases assisted by the ResAM50 deep learning model [86]: (i) A 34-year-old female with a breast lesion detected on US. PA-US imaging showed oxygenation signals around the lesion but none inside. Pathology confirmed a fibroadenoma; (ii) A 64-year-old female with a lesion seen on US. PA-US imaging revealed oxygenation signals both inside and around the lesion. Pathology confirmed invasive ductal carcinoma. (**c**) Learning-based classification, localization, and segmentation of breast lesions [90]. (i) Each whole-breast PACT image of the patient was rendered as a 2D MAP and divided into four quadrants. (ii) Image processing, feature extraction, and lesion classification and segmentation. (**d**) Imaging results of a breast cancer patient [56]: (i) A fused image of PA and 3D ultrasound (3D-US). The US data were colored red. Angiogenesis were occurred near the tumor; (ii) A fusion of the S-factor image and 3D-US image (red). The nipple appears light blue due to the spectral similarity between melanin absorption and low sO_2_. The S-factor indicates the correlation between true and measured sO_2_. (**e**) PA-US fusion images [64]: Imagio^®^ system image showing high deoxyhemoglobin levels inside the tumor. Rich vasculature is also visible at the tumor margin (arrow), including radial arteries (green) and veins (red); (**f**) An image from a handheld PA-US system, showing breast tumor sO_2_ mapping [66]. (**g**) Imaging of an inflammatory breast cancer case using the PAM-03 prototype demonstrated a lesion with a diameter of 47 mm [55]: (i) Contrast-enhanced MRI with the lesion circled in red; (ii) Original PA image; (iii) Fused image of the PA image (cyan) and the MRI image (red).

In the clinical study led by Xia’s group, 38 biopsy-confirmed breast cancer patients were recruited, and both the affected and contralateral breasts were imaged using self-developed system. Due to elevated vascular density, tumor regions exhibited strong photoacoustic signals, enabling accurate localization of the lesions. Furthermore, as shown in Figure 3a, the study based on the PAM-2 system developed by Manohar’s group revealed speckled photoacoustic signals within tumor regions. These signals were considered helpful in distinguishing between invasive carcinoma and ductal carcinoma in situ. Other clinical studies have demonstrated that the level and distribution of oxygen saturation in the tumor microenvironment differ significantly between malignant and benign breast lesions, confirming the diagnostic value of functional PAI parameters for breast cancer [87,88,89].

Besides the tumor interior, multiple studies on breast PA functional imaging have revealed differences in PA signal distribution at the margins between benign and malignant breast lesions [89,91,92]. Specifically, the mean sO_2_ inside the T1-stage invasive breast cancer tumor was lower than that of benign tumors and healthy breast tissue [92]. In the tumor margin region, the mean value of sO_2_ of T1-stage invasive breast cancer was 4.9% lower than that of benign tumors, and the vasculature surrounding malignant lesions exhibited a more diffuse distribution [91]. These finding provide strong functional imaging evidence for distinguishing between benign and malignant breast tumors, highlighting the broad clinical potential of PAI in accurate and noninvasive breast cancer diagnosis.

Deep learning has become a key tool for medical image analysis. Generative adversarial networks (GANs) have been successfully applied to a wide range of tasks such as medical image generation [93,94], denoising [95,96], reconstruction [97], segmentation [98], detection [99] and classification [100]. In order to further enhance the feature selection ability of GANs, Zhang et al. [101] introduced the self-attention mechanism into GANs in 2019. Subsequently, the Transformer model [102] performed well in visual tasks due to its ability to handle long sequence data and improve training speed. Jiang et al. [103] built a convolution-free GAN model (TransGAN) with pure Transformer architecture for the first time. Meanwhile, researchers also embedded Transformers into the GAN framework for specific medical tasks. For example, Luo et al. [104] used a Transformer-GAN to reconstruct standard dose PET images from low-dose PET images. GAN based on Transformer shows great potential in the field of medical image analysis. In breast photoacoustic imaging, deep learning plays an important role in data generation, image reconstruction and diagnostic analysis. In order to solve the problem of lack of clinical PAT data, Ma et al. and Lou et al. automatically built a breast numerical model with real anatomical structure (such as vascular distribution) and optical/acoustic properties based on mammography images [105] or clinical MRI data [106], providing reliable data support for simulation research. In order to solve the quality and efficiency of PAT image reconstruction from a limited perspective, John et al. [107] developed a non-iterative deep learning network (U-SBTV) based on SBTV and RBP ADMM algorithm. Combined with transfer learning and U-Net denoiser, it significantly improved the image quality (MS-SSIM > 0.95) and efficiency (the reconstruction time was shortened by 92%), and verified the effectiveness of the semi-circular array through theoretical analysis. In diagnostic aspect, a deep learning algorithm based on the ResNet50 model and integrated with an attention mechanism has been proposed to enhance breast cancer prediction accuracy by analyzing PA-US images [86]. In a study involving 334 breast cancer patients, the model significantly increased diagnostic specificity compared to traditional methods, without compromising sensitivity, demonstrating promising potential for clinical application (Figure 3b). Tong et al. analyzed PACT features from 78 breasts of 39 patients and developed machine learning classifiers to distinguish between normal and suspicious tissues (Figure 3c), achieving a maximum area under the curve (AUC) of 0.89—comparable to that of conventional imaging modalities [90]. In addition, 13 quantitative image features were extracted and utilized to further differentiate malignant from benign lesions. The authors also proposed a learning-based model for automated lesion segmentation, which incorporated manually annotated lesion boundaries as training labels. These findings underscore the potential of PACT as a noninvasive and sensitive imaging modality for breast lesion characterization.

In addition to algorithmic optimization, image fusion techniques have also contributed to improved screening and diagnostic accuracy. A clinical evaluation of the PAI-04 system, developed by Tsuyoshi Shiina’s team, demonstrated that multimodal fusion enhances diagnostic performance. As shown in Figure 3d, the fused image combining photoacoustic and 3D ultrasound modalities clearly visualizes the vascular structures surrounding the tumor [56].

Multiple large-scale clinical studies utilizing the Imagio^®^ breast imaging system have demonstrated improved diagnostic accuracy through the integration of PA and US image features. In the PIONEER study, the system was used to image 1690 patients with breast tumors, evaluating 1757 lesions (1079 malignant and 678 benign) categorized as BI-RADS 3, 4, or 5 (Figure 3e) [63,64]. The results showed that PA-US fusion imaging improved diagnostic specificity by 14.9% compared to US alone [63]. In a subsequent analysis, 532 breast cancers from 519 women were examined to assess the system’s ability to differentiate molecular subtypes. Statistically significant differences in PA and US image features were observed between luminal (A and B) subtypes and other subtypes such as triple-negative and HER2-positive cancers. Another study involving 215 breast masses from 209 patients demonstrated the system’s potential to refine BI-RADS classification and enhance diagnostic precision [91]. Specifically, benign lesions initially classified as BI-RADS 4a on conventional US were successfully downgraded based on PA imaging features. Five key PA image features were scored and weighted to reassess malignancy probability, differentiate molecular subtypes, and adjust BI-RADS categories. These included: (1) Intratumoral blood vessel density and deoxyhemoglobin content; (2) Blush and deoxyhemoglobin content within the mass; (3) Total hemoglobin concentration; (4) Vascular distribution and deoxyhemoglobin content at the tumor margins; (5) Radial vascular patterns in the peritumoral region.

In addition, the handheld device developed by Li’s team [50,66] enables dual-modality PA/US imaging. A preliminary clinical evaluation involving 1 healthy volunteer and 2 patients with benign tumors was conducted to verify the device’s accuracy in measuring breast tissue sO_2_, as shown in Figure 3f. In another clinical study, the DSM system developed by Xia’s team was used to image 38 biopsy-confirmed patients with various breast tumor types [82]. The results demonstrated the system’s ability to differentiate tumor-bearing breasts from healthy ones, with tumor regions exhibiting larger-caliber blood vessels and greater background signal heterogeneity. Preliminary findings also suggested its potential in distinguishing between different tumor subtypes. These results further support the clinical value of combining PA and US imaging to improve breast cancer screening and diagnosis.

In addition to fusion with US, PAI combined with MRI offers notable advantages. The third-generation breast PAI system, PAM-03, developed by Kyoto University and Canon Inc. The clinical studies have demonstrated that the system clearly visualizes centripetal vascular in invasive breast cancer, such as abrupt vessel narrowing or peripheral rupture, which differs from the vascular features of ductal carcinoma in situ (DCIS) [55,108]. Compared to MRI, the fusion PA/MRI images provide finer vascular details, more complete branching structures, and richer morphological information (Figure 3g) [55]. These results suggest that PAI holds strong potential for noninvasive vascular assessment and may complement MRI in improving early breast cancer diagnosis.

## 4. Evaluation of Breast Cancer Treatment

As an extension of early screening and accurate diagnosis, accurate treatment of breast cancer has become a key link in clinical practice. However, there is a significant disconnection in the current diagnosis and treatment system. For example, the lack of dynamic monitoring means in the treatment process seriously restricts the optimization and implementation of individualized programs. In order to break through this bottleneck, multiple PAI research teams carry out systematic exploration in the three major areas of neoadjuvant therapy efficacy evaluation, intraoperative resection margin detection and drug delivery monitoring, to promote the establishment of integration of diagnosis and treatment.

**Figure 4:**
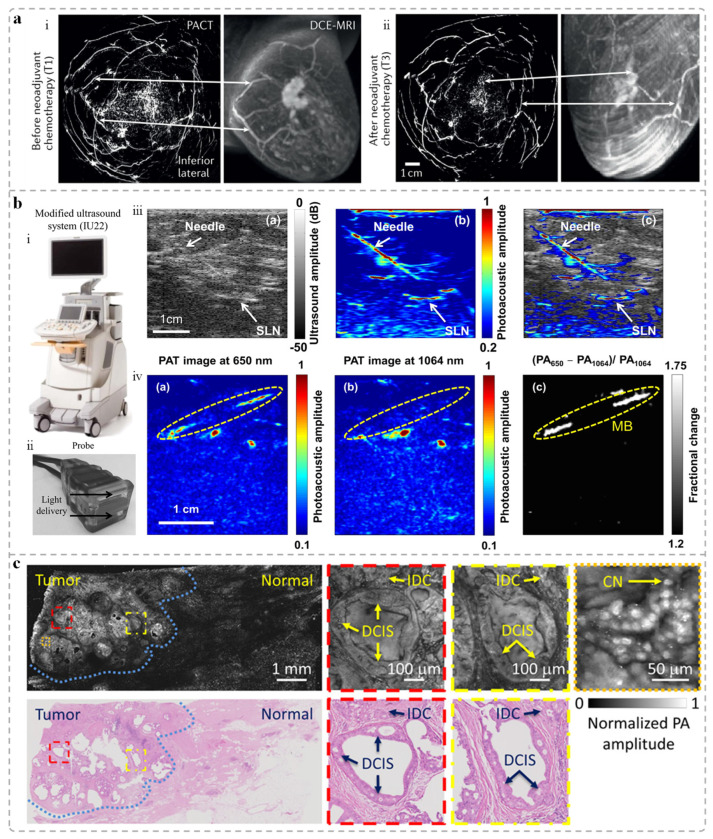
Evaluation of PAI therapeutic efficacy in breast tumors. (**a**) Comparison of the images acquired by the SBH-PACT and contrast-enhanced MRI [110]. (i) and (ii) represent the PACT images (left, imaging time 15 s without contrast injection) and the contrast-enhanced MRI images (right) of the same breast before (T1) and after (T3) neoadjuvant chemotherapy, respectively. Related structures are marked with white arrows. (**b**) Dual-modal PA-US imaging system and sentinel lymph node (SLN) imaging results [113]: (i) Photograph of the dual-modal PA-US imaging system; (ii) Photograph of the handheld probe; (iii) Real-time in vivo imaging during SLN biopsy: US image with the lymph node and needle, showing low contrast of them (left); The PA image with the SLN and needle, which are clearly visualized (middle); The co-registered PA-US image with the alignment of the SLN and needle (right); (iv) In vivo imaging of methylene blue and its differentiation from blood vessels: PA image at 650 nm with signals from both methylene blue and hemoglobin (left); PA image at 1064 nm with hemoglobin signals (middle); Image highlighting the distribution of methylene blue (right). (**c**) Images of a patient breast tumor [44]. Top tow: The UV-PAM images of a unsliced breast tumor and magnified views of the boxed areas. Bottom row: H&E-stained histologic image of the same regions in the breast tumor and magnified views of the boxed areas. The blue dotted line represents the boundary between normal tissue and tumor.

In the field of neoadjuvant therapy monitoring, traditional imaging technologies are difficult to accurately reflect the treatment response and rapid evaluation of the effectiveness of therapy, due to only the morphological evaluation and the presence of interference from tumor fibrosis [109]. The SBH-PACT system developed by Wang’s team completed a PA scanning imaging before, during, and after neoadjuvant chemotherapy (NAC) (Figure 4a) [110]. By extracting the PA image features related to blood vessel density, distribution and morphology of the lesion, the results of comparing with histopathology reveals that PA image information has the potential to accurately evaluate the efficacy of NAC, and can realize the noninvasive evaluation of cases in the future. Angiogenesis and sO_2_ within breast tumor and its the boundary zone are associated with tumor development and prognosis [111,112]. The breast clinical is expecting with a reliable and imaging clearer PAI system to conduct large-scale clinical studies, finding the relationship with PA image features and pathological results. Finally, PAI can integrate into the clinical workflow of neoadjuvant therapy.

In addition to medical treatment represented by neoadjuvant therapy, surgery is also an important method of breast cancer treatment. During surgery, sentinel lymph node (SLN) biopsy is important to assess breast cancer staging, and the result directly influences prognosis evaluation and treatment planning. Due to the low contrast of lymph node with ultrasound [114], the clinic necessitates lymph node excision to ensure diagnostic accuracy, increasing surgical risks for patients [115]. Wang’s group developed a PA/US dual-modality imaging system, showing great potential in intraoperative guidance (Figure 4b) [113]. This system enables noninvasive visualization of methylene blue dye within SLNs, significantly improving imaging contrast and lymph node detectability. The system allows real-time tracking of needle insertion paths (Figure 4b), providing precise guidance for biopsy procedures [116,117]. Figure 4b also illustrates the system’s ability to distinguish methylene blue distribution from surrounding vasculature in vivo, further supporting its effectiveness for SLN localization.

Whether tumor tissue has been completely removed during surgery is typically assessed using intraoperative frozen section analysis. However, it is time-consuming (around 30 min) and highly dependent on the pathologist’s expertise. Consequently, 20–40% of breast cancer patients still require reoperation following initial surgery [118]. To address this limitation, researchers are actively investigating PAI as a next-generation intraoperative margin assessment tool. Ultraviolet photoacoustic microscopy (UV-PAM) leverages the endogenous absorption properties of DNA and RNA in the ultraviolet spectrum to generate tissue contrast comparable to that of hematoxylin and eosin (H&E) staining, without the need for exogenous dyes (Figure 4c) [44]. This significantly simplifies tissue preparation and expedites analysis, though it still requires tissue sectioning. To enhance imaging speed, microlenses and linear ultrasound arrays have been incorporated, enabling parallel excitation and signal detection [119]. With the integration of rapid scanning and computer-aided diagnosis, UV-PAM shows potential for real-time intraoperative histopathological evaluation. In addition, to improve imaging specificity, some studies have introduced multi-wavelength illumination to extract richer tissue information. For instance, Kim et al. implemented 700 nm excitation for effective imaging of breast microcalcifications [120], broadening the application of PA technology in the diagnosis of breast lesions.

In addition, exogenous contrast agents can be used to enhance PA signals of tumor. Grootendorst’s team developed a clinically validated, MRI-compatible PA probe, which based on magnetic iron oxide nanoparticles [121]. Jiang’s group engineered peptide-conjugated nanoparticles carrying near-infrared (NIR) dyes, achieving targeted imaging with high contrast and strong penetration in a murine breast cancer model [122]. Although none of the PAI probe have yet been approved by the FDA, numerous contrast agents with strong absorption in specific spectral bands have shown considerable potential for future clinical application [123].

Real-time monitoring of pharmacokinetics and pharmacodynamics in biological tissues is crucial for evaluating tumor treatment. With high contrast, high resolution, substantial imaging depth, and rapid imaging speed, PAI offers distinct advantages over conventional imaging modalities in small animal studies [124,125]. For instance, PAI enables real-time tracking of photothermal agents and photosensitizers, providing precise feedback for photothermal therapy and photodynamic therapy [125]. However, to address the challenge of weak optical absorption in some therapeutic drugs, multiple research teams have developed a variety of NIR absorbance-enhanced drugs carry to improve PA contrast [126]. For example, doxorubicin, which can inhibit the proliferation of human breast cancer cell lines, can be encapsulated in gold nanoparticles, and then the dynamic monitoring of breast cancer cell lines treatment can be achieved by PAI [127]. PAI, which can provide high-resolution, fast and rich contrast local imaging, has the potential to supplement positron emission tomography, although it is still in early stage in the field of drug delivery monitoring.

## 5. Discussion and Conclusion

In recent years, PAI has demonstrated significant potential in the early screening, accurate diagnosis, and treatment evaluation of breast tumors. Multiple research teams have developed a variety of PAI systems for breast imaging, utilizing customized ultrasound transducer arrays to acquire tumor-associated features such as hemoglobin concentration, sO_2_, and vascular architecture. Some systems integrate US and PAI modalities, enabling the simultaneous acquisition of anatomical and functional information within a unified coordinate system, thereby improving clinical efficiency.

Clinical studies have shown that oxygenation levels and vascular distribution within the tumor microenvironment can assist in differentiating benign from malignant lesions, classifying molecular subtypes, and refining BI-RADS categorization. For treatment evaluation, PAI enables real-time monitoring of changes in the tumor microenvironment, aiding in the assessment of therapeutic response and prognosis. In intraoperative applications, PAI shows promise for tumor margin identification and rapid margin assessment. Furthermore, PAI may enhance the accuracy of sentinel lymph node (SLN) biopsy by compensating for the limited contrast of conventional US. When combined with exogenous contrast agents or molecular probes, PAI demonstrates strong potential for breast cancer–specific detection. Although many of these applications remain in the preclinical stage, the prospects for clinical translation are encouraging. In 2021, the U.S. Food and Drug Administration (FDA) approved the first clinical breast PAI device, Imagio^®^, marking a milestone in the clinical adoption of PAI.

Nevertheless, several challenges remain before widespread clinical implementation can be achieved. Systems developed by different manufacturers and research groups adopt non-standardized phantoms and methods to verify the performance of the system, which makes it impossible to compare the performance of different instruments due to differences in image quality and interpretation. Even for the same equipment, there is no standardized phantom or method in the preclinical test phase, which leads to unexpected results in experiments across different batches. In clinical tests, unified image quality cannot be guaranteed among patients with large individual differences. This undoubtedly reduces the repeatability of experimental operations and results. Although the technology and equipment indicators developed by laboratories are advanced, a large number of experiments and data processing are required. The image quality of products developed by companies is relatively poor, which can-not reflect the advancement and irreplaceability of photoacoustic imaging technology. Therefore, the establishment of PAI standardization is conducive to systematic comparison between different devices and repeatable operation of the same device, and can accelerate the clinical translation process of photoacoustic imaging systems.

In addition, in order to ensure the reliability of clinical testing, it is also necessary to improve the design and manufacture of products at the system engineering level to avoid interference from multiple factors in the results [128]. For example, the sensitivity of optical components to dust and contamination, changes in the clinical testing environment caused by high-energy laser output, and the demands for specialized equipment and technical personnel training. PAI equipment for clinical use must be strictly reviewed and approved by relevant institutions. Large-scale clinical validation is needed to ensure safety, accuracy, and clinical practicability [129]. Therefore, regulatory approval is an important part of clinical translation. At present, large-scale clinical validation is insufficient, and only a few PAI platforms have been approved. Representative photoacoustic devices include the Imagio^®^ Breast Imaging System and the Acuity Echo^®^ system.

Over the past decade, PAI has matured considerably in small animal imaging, offering high spatial resolution and deep tissue penetration, and enabling multi-scale imaging from cellular to organ levels. However, clinical translation in breast imaging largely remains at the feasibility study stage. As an emerging medical imaging modality, PAI—especially when integrated with US—has the potential to rapidly and accurately assess both anatomical and functional features of the breast tumor microenvironment, providing valuable clinical information. To fully establish the clinical utility of photoacoustic imaging (PAI), it is essential to shift research from small-scale exploratory studies to large-sample, multicenter clinical trials that support its integration into routine diagnostic and therapeutic work-flows. In parallel, since its establishment in 2018 [130], the International Photoacoustic Standardisation Consortium (IPASC) has been driving key standardization initiatives. These efforts include developing a unified data format with consensus metadata structures and open-source tools for data conversion to promote interoperability across platforms. Moreover, IPASC has introduced standardized terminology, performance metrics, and a comprehensive library of image reconstruction algorithms to enable reproducible and consistent image analysis [131].

## Data Availability

No new data were created or analyzed in this study. Data sharing is not applicable to this article.

## References

[1] Ren W., Chen M., Qiao Y., Zhao F. (2022). Global Guidelines for Breast Cancer Screening: A Systematic Review. Breast.

[2] Kim J., Harper A., McCormack V., Sung H., Houssami N., Morgan E., Mutebi M., Garvey G., Soerjomataram I., Fidler-Benaoudia M.M. (2025). Global Patterns and Trends in Breast Cancer Incidence and Mortality across 185 Countries. Nat. Med..

[3] Beckmann M., Niederacher D., Schnürch H.-G., Gusterson B.A., Bender H.G. (1997). Multistep Carcinogenesis of Breast Cancer and Tumour Heterogeneity. J. Mol. Med..

[4] Nik-Zainal S., Davies H., Staaf J., Ramakrishna M., Glodzik D., Zou X., Martincorena I., Alexandrov L.B., Martin S., Wedge D.C. (2016). Landscape of Somatic Mutations in 560 Breast Cancer Whole-Genome Sequences. Nature.

[5] Nolan E., Lindeman G.J., Visvader J.E. (2023). Deciphering Breast Cancer: From Biology to the Clinic. Cell.

[6] Bertucci F., Finetti P., Birnbaum D. (2012). Basal Breast Cancer: A Complex and Deadly Molecular Subtype. Curr. Mol. Med..

[7] Marvalim C., Datta A., Lee S.C. (2023). Role of P53 in Breast Cancer Progression: An Insight into P53 Targeted Therapy. Theranostics.

[8] Yersal O., Barutca S. (2014). Biological Subtypes of Breast Cancer: Prognostic and Therapeutic Implications. World J. Clin. Oncol..

[9] Vaupel P., Multhoff G. (2018). Hypoxia-/HIF-1α-Driven Factors of the Tumor Microenvironment Impeding Antitumor Immune Responses and Promoting Malignant Progression. Oxygen Transport to Tissue XL.

[10] Jamil A., Qureshi Z., Amankwah M. (2025). Dual Inhibition of HER2 and VEGF Pathways in Breast Cancer: A Meta-Analysis of Outcomes. J. Clin. Oncol..

[11] CW E. (1991). The Value of Histological Grade in Breast Cancer: Experience from a Large Study with Long-Term Follow-Up. Histopathology.

[12] Badowska-Kozakiewicz A., Sobol M., Patera J., Kozłowski W. (2013). Immunohistochemical Evaluation of Human Epidermal Growth Factor Receptor 2 and Estrogen and Progesterone Receptors in Invasive Breast Cancer in Women. Arch. Med. Sci..

[13] Rosen P.P. (2001). Rosen’s Breast Pathology.

[14] Badowska-Kozakiewicz A., Liszcz A., Sobol M., Patera J. (2017). Retrospective Evaluation of Histopathological Examinations in Invasive Ductal Breast Cancer of No Special Type: An Analysis of 691 Patients. Arch. Med. Sci..

[15] Turajlic S., Sottoriva A., Graham T., Swanton C. (2019). Resolving Genetic Heterogeneity in Cancer. Nat. Rev. Genet..

[16] Delaloge S., Khan S.A., Wesseling J., Whelan T. (2024). Ductal Carcinoma in Situ of the Breast: Finding the Balance between Overtreatment and Undertreatment. Lancet.

[17] Abdelrahman L., Al Ghamdi M., Collado-Mesa F., Abdel-Mottaleb M. (2021). Convolutional Neural Networks for Breast Cancer Detection in Mammography: A Survey. Comput. Biol. Med..

[18] Loizidou K., Elia R., Pitris C. (2023). Computer-Aided Breast Cancer Detection and Classification in Mammography: A Comprehensive Review. Comput. Biol. Med..

[19] Aberle D.R., Chiles C., Gatsonis C., Hillman B.J., Johnson C.D., McClennan B.L., Mitchell D.G., Pisano E.D., Schnall M.D., Sorensen A.G. (2005). Imaging and Cancer: Research Strategy of the American College of Radiology Imaging Network. Radiology.

[20] Leung J.W. (2005). Screening Mammography Reduces Morbidity of Breast Cancer Treatment. Am. J. Roentgenol..

[21] Colin C., de Vathaire F., Noël A., Charlot M., Devic C., Foray N., Valette P.-J. (2012). Updated Relevance of Mammographic Screening Modalities in Women Previously Treated with Chest Irradiation for Hodgkin Disease. Radiology.

[22] Tokunaga M. (1978). Breast Cancer among Atomic Bomb Survivors.

[23] Little M. (2001). Comparison of the Risks of Cancer Incidence and Mortality Following Radiation Therapy for Benign and Malignant Disease with the Cancer Risks Observed in the Japanese A-Bomb Survivors. Int. J. Radiat. Biol..

[24] Henderson T.O., Amsterdam A., Bhatia S., Hudson M.M., Meadows A.T., Neglia J.P., Diller L.R., Constine L.S., Smith R.A., Mahoney M.C. (2010). Systematic Review: Surveillance for Breast Cancer in Women Treated with Chest Radiation for Childhood, Adolescent, or Young Adult Cancer. Ann. Intern. Med..

[25] Sharma N., McMahon M., Haigh I., Chen Y., Dall B.J. (2019). The Potential Impact of Digital Breast Tomosynthesis on the Benign Biopsy Rate in Women Recalled within the UK Breast Screening Programme. Radiology.

[26] Nayak K.S., Lim Y., Campbell-Washburn A.E., Steeden J. (2022). Real-time Magnetic Resonance Imaging. J. Magn. Reson. Imaging.

[27] Lin L., Wang L.V., Wei X., Gu B. (2021). Photoacoustic Imaging. Optical Imaging in Human Disease and Biological Research.

[28] Zangabad R.P., Iskander-Rizk S., van der Meulen P., Meijlink B., Kooiman K., Wang T., van der Steen A.F., van Soest G. (2021). Photoacoustic Flow Velocity Imaging Based on Complex Field Decorrelation. Photoacoustics.

[29] Ahn J., Kim J.Y., Choi W., Kim C. (2021). High-Resolution Functional Photoacoustic Monitoring of Vascular Dynamics in Human Fingers. Photoacoustics.

[30] Lv J., Lan H., Qin A., Sun T., Shao D., Gao F., Yao J., Avanaki K., Nie L. (2024). Dynamic Synthetic-Scanning Photoacoustic Tracking Monitors Hepatic and Renal Clearance Pathway of Exogeneous Probes in vivo. Light Sci. Appl..

[31] Cao Y., Dumani D.S., Hallam K.A., Emelianov S.Y., Ran H. (2023). Real-Time Monitoring of NIR-Triggered Drug Release from Phase-Changeable Nanodroplets by Photoacoustic/Ultrasound Imaging. Photoacoustics.

[32] Chu C., Yu J., Ren E., Ou S., Zhang Y., Wu Y., Wu H., Zhang Y., Zhu J., Dai Q. (2020). Multimodal Photoacoustic Imaging-Guided Regression of Corneal Neovascularization: A Non-Invasive and Safe Strategy. Adv. Sci..

[33] Lin L., Wang L.V. (2022). The Emerging Role of Photoacoustic Imaging in Clinical Oncology. Nat. Rev. Clin. Oncol..

[34] Qiu T., Lan Y., Gao W., Zhou M., Liu S., Huang W., Zeng S., Pathak J.L., Yang B., Zhang J. (2021). Photoacoustic Imaging as a Highly Efficient and Precise Imaging Strategy for the Evaluation of Brain Diseases. Quant. Imaging Med. Surg..

[35] Jo J., Tian C., Xu G., Sarazin J., Schiopu E., Gandikota G., Wang X. (2018). Photoacoustic Tomography for Human Musculoskeletal Imaging and Inflammatory Arthritis Detection. Photoacoustics.

[36] Lao Y., Xing D., Yang S., Xiang L. (2008). Noninvasive Photoacoustic Imaging of the Developing Vasculature during Early Tumor Growth. Phys. Med. Biol..

[37] Liu S., Zhang R., Han T., Pan Y., Zhang G., Long X., Zhao C., Wang M., Li X., Yang F. (2022). Validation of Photoacoustic/Ultrasound Dual Imaging in Evaluating Blood Oxygen Saturation. Biomed. Opt. Express.

[38] Laufer J., Johnson P., Zhang E., Treeby B., Cox B., Pedley B., Beard P. (2012). In Vivo Preclinical Photoacoustic Imaging of Tumor Vasculature Development and Therapy. J. Biomed. Opt..

[39] Yao J., Wang L.V. (2013). Photoacoustic Microscopy. Laser Photonics Rev..

[40] Guo H., Li Y., Qi W., Xi L. (2020). Photoacoustic Endoscopy: A Progress Review. J. Biophotonics.

[41] Xia J., Yao J., Wang L.V. (2014). Photoacoustic Tomography: Principles and Advances. Electromagn. Waves.

[42] Attia A.B.E., Chuah S.Y., Razansky D., Ho C.J.H., Malempati P., Dinish U., Bi R., Fu C.Y., Ford S.J., Lee J.S.-S. (2017). Noninvasive Real-Time Characterization of Non-Melanoma Skin Cancers with Handheld Optoacoustic Probes. Photoacoustics.

[43] Li D., Humayun L., Vienneau E., Vu T., Yao J. (2021). Seeing through the Skin: Photoacoustic Tomography of Skin Vasculature and Beyond. JID Innov..

[44] Wong T.T., Zhang R., Hai P., Zhang C., Pleitez M.A., Aft R.L., Novack D.V., Wang L.V. (2017). Fast Label-Free Multilayered Histology-like Imaging of Human Breast Cancer by Photoacoustic Microscopy. Sci. Adv..

[45] Wu Z., Li L., Yang Y., Hu P., Li Y., Yang S.-Y., Wang L.V., Gao W. (2019). A Microrobotic System Guided by Photoacoustic Computed Tomography for Targeted Navigation in Intestines in Vivo. Sci. Robot..

[46] Kothapalli S.-R., Sonn G.A., Choe J.W., Nikoozadeh A., Bhuyan A., Park K.K., Cristman P., Fan R., Moini A., Lee B.C. (2019). Simultaneous Transrectal Ultrasound and Photoacoustic Human Prostate Imaging. Sci. Transl. Med..

[47] Rao A.P., Bokde N., Sinha S. (2020). Photoacoustic Imaging for Management of Breast Cancer: A Literature Review and Future Perspectives. Appl. Sci..

[48] Lin L., Hu P., Shi J., Appleton C.M., Maslov K., Li L., Zhang R., Wang L.V. (2018). Single-Breath-Hold Photoacoustic Computed Tomography of the Breast. Nat. Commun..

[49] Nyayapathi N., Xia J. (2019). Photoacoustic Imaging of Breast Cancer: A Mini Review of System Design and Image Features. J. Biomed. Opt..

[50] Zhang G., Li W., Yang M., Li C. (2020). Developing a Photoacoustic Whole-Breast Imaging System Based on the Synthetic Matrix Array. Front. Phys..

[51] Lin L., Hu P., Tong X., Na S., Cao R., Yuan X., Garrett D.C., Shi J., Maslov K., Wang L.V. (2021). High-Speed Three-Dimensional Photoacoustic Computed Tomography for Preclinical Research and Clinical Translation. Nat Commun.

[52] Shiina T., Toi M., Yagi T. (2018). Development and Clinical Translation of Photoacoustic Mammography. Biomed. Eng. Lett..

[53] Fukutani K., Someda Y., Taku M., Asao Y., Kobayashi S., Yagi T., Yamakawa M., Shiina T., Sugie T., Toi M. (2011). Characterization of Photoacoustic Tomography System with Dual Illumination.

[54] Asao Y., Hashizume Y., Suita T., Nagae K., Fukutani K., Sudo Y., Matsushita T., Kobayashi S., Tokiwa M., Yamaga I. (2016). Photoacoustic Mammography Capable of Simultaneously Acquiring Photoacoustic and Ultrasound Images. J. Biomed. Opt..

[55] Toi M., Asao Y., Matsumoto Y., Sekiguchi H., Yoshikawa A., Takada M., Kataoka M., Endo T., Kawaguchi-Sakita N., Kawashima M. (2017). Visualization of Tumor-Related Blood Vessels in Human Breast by Photoacoustic Imaging System with a Hemispherical Detector Array. Sci. Rep..

[56] Matsumoto Y., Asao Y., Sekiguchi H., Yoshikawa A., Ishii T., Nagae K., Kobayashi S., Tsuge I., Saito S., Takada M. (2018). Visualising Peripheral Arterioles and Venules through High-Resolution and Large-Area Photoacoustic Imaging. Sci. Rep..

[57] Schoustra S.M., Piras D., Huijink R., op ‘t Root T.J., Alink L., Kobold W.M., Steenbergen W., Manohar S. (2019). Twente Photoacoustic Mammoscope 2: System Overview and Three-Dimensional Vascular Network Images in Healthy Breasts. J. Biomed. Opt..

[58] Dantuma M., Lucka F., Kruitwagen S., Javaherian A., Alink L., van Meerdervoort R., Nanninga M., Root T., De Santi B., Budisky J. (2023). Fully Three-Dimensional Sound Speed-Corrected Multi-Wavelength Photoacoustic Breast Tomography. arXiv.

[59] Matsumoto Y., Asao Y., Yoshikawa A., Sekiguchi H., Takada M., Furu M., Saito S., Kataoka M., Abe H., Yagi T. (2018). Label-Free Photoacoustic Imaging of Human Palmar Vessels: A Structural Morphological Analysis. Sci. Rep..

[60] Sato Y., Shiraga N., Nakajima S., Tamura S., Kikinis R. (1998). Local Maximum Intensity Projection (LMIP: A New Rendering Method for Vascular Visualization. J. Comput. Assist. Tomogr..

[61] Schoustra S.M., De Santi B., op’t Root T.J., Klazen C.A., van der Schaaf M., Veltman J., Steenbergen W., Manohar S. (2023). Imaging Breast Malignancies with the Twente Photoacoustic Mammoscope 2. PLoS ONE.

[62] Stephens K. (2021). FDA Approves Seno Medical’s Breast Cancer Diagnostic Technology. AXIS Imaging News.

[63] Neuschler E.I., Butler R., Young C.A., Barke L.D., Bertrand M.L., Böhm-Vélez M., Destounis S., Donlan P., Grobmyer S.R., Katzen J. (2018). A Pivotal Study of Optoacoustic Imaging to Diagnose Benign and Malignant Breast Masses: A New Evaluation Tool for Radiologists. Radiology.

[64] Oraevsky A., Clingman B., Zalev J., Stavros A., Yang W., Parikh J. (2018). Clinical Optoacoustic Imaging Combined with Ultrasound for Coregistered Functional and Anatomical Mapping of Breast Tumors. Photoacoustics.

[65] Diot G., Metz S., Noske A., Liapis E., Schroeder B., Ovsepian S.V., Meier R., Rummeny E., Ntziachristos V. (2017). Multispectral Optoacoustic Tomography (MSOT) of Human Breast Cancer. Clin. Cancer Res..

[66] Han T., Yang M., Yang F., Zhao L., Jiang Y., Li C. (2021). A Three-Dimensional Modeling Method for Quantitative Photoacoustic Breast Imaging with Handheld Probe. Photoacoustics.

[67] Deán-Ben X.L., Fehm T.F., Gostic M., Razansky D. (2016). Volumetric Hand-held Optoacoustic Angiography as a Tool for Real-time Screening of Dense Breast. J. Biophotonics.

[68] Riksen J.J., Nikolaev A.V., van Soest G. (2023). Photoacoustic Imaging on Its Way toward Clinical Utility: A Tutorial Review Focusing on Practical Application in Medicine. J. Biomed. Opt..

[69] Goh Y., Balasundaram G., Tan H.M., Putti T.C., Renzhe B., Hartman M., Buhari S.A., Ng C.W.Q., Lui S.A., Fang E. (2024). Ultrasound-Guided Photoacoustic (US-PA) Tomography of the Breast: Biochemical Differentiation Using Intrinsic Tissue Markers—Lipids, Collagen and Hemoglobin with Histopathologic Correlation. Sci. Rep..

[70] Lee C., Kim C., Park B. (2023). Review of Three-Dimensional Handheld Photoacoustic and Ultrasound Imaging Systems and Their Applications. Sensors.

[71] Stoffels I., Morscher S., Helfrich I., Hillen U., Leyh J., Burton N.C., Sardella T.C., Claussen J., Poeppel T.D., Bachmann H.S. (2015). Metastatic Status of Sentinel Lymph Nodes in Melanoma Determined Noninvasively with Multispectral Optoacoustic Imaging. Sci. Transl. Med..

[72] Knieling F., Neufert C., Hartmann A., Claussen J., Urich A., Egger C., Vetter M., Fischer S., Pfeifer L., Hagel A. (2017). Multispectral Optoacoustic Tomography for Assessment of Crohn’s Disease Activity. N. Engl. J. Med..

[73] Reber J., Willershäuser M., Karlas A., Paul-Yuan K., Diot G., Franz D., Fromme T., Ovsepian S.V., Bézière N., Dubikovskaya E. (2018). Non-Invasive Measurement of Brown Fat Metabolism Based on Optoacoustic Imaging of Hemoglobin Gradients. Cell Metab..

[74] Li Y., Schnabl K., Gabler S.-M., Willershäuser M., Reber J., Karlas A., Laurila S., Lahesmaa M., Din M.u., Bast-Habersbrunner A. (2018). Secretin-Activated Brown Fat Mediates Prandial Thermogenesis to Induce Satiation. Cell.

[75] Yang H., Jüstel D., Prakash J., Karlas A., Helfen A., Masthoff M., Wildgruber M., Ntziachristos V. (2020). Soft Ultrasound Priors in Optoacoustic Reconstruction: Improving Clinical Vascular Imaging. Photoacoustics.

[76] Masthoff M., Helfen A., Claussen J., Karlas A., Markwardt N.A., Ntziachristos V., Eisenblätter M., Wildgruber M. (2018). Use of Multispectral Optoacoustic Tomography to Diagnose Vascular Malformations. JAMA Dermatol..

[77] Roll W., Markwardt N.A., Masthoff M., Helfen A., Claussen J., Eisenblätter M., Hasenbach A., Hermann S., Karlas A., Wildgruber M. (2019). Multispectral Optoacoustic Tomography of Benign and Malignant Thyroid Disorders: A Pilot Study. J. Nucl. Med..

[78] Masthoff M., Helfen A., Claussen J., Roll W., Karlas A., Becker H., Gabriëls G., Riess J., Heindel W., Schäfers M. (2018). Multispectral Optoacoustic Tomography of Systemic Sclerosis. J. Biophotonics.

[79] Regensburger A.P., Fonteyne L.M., Jüngert J., Wagner A.L., Gerhalter T., Nagel A.M., Heiss R., Flenkenthaler F., Qurashi M., Neurath M.F. (2019). Detection of Collagens by Multispectral Optoacoustic Tomography as an Imaging Biomarker for Duchenne Muscular Dystrophy. Nat. Med..

[80] Kukačka J., Metz S., Dehner C., Muckenhuber A., Paul-Yuan K., Karlas A., Fallenberg E.M., Rummeny E., Jüstel D., Ntziachristos V. (2022). Image Processing Improvements Afford Second-Generation Handheld Optoacoustic Imaging of Breast Cancer Patients. Photoacoustics.

[81] Nyayapathi N., Lim R., Zhang H., Zheng W., Wang Y., Tiao M., Oh K.W., Fan X.C., Bonaccio E., Takabe K. (2020). Dual Scan Mammoscope (DSM)—A New Portable Photoacoustic Breast Imaging System With Scanning in Craniocaudal Plane. IEEE Trans. Biomed. Eng..

[82] Nyayapathi N., Zhang H., Zheng E., Nagarajan S., Bonaccio E., Takabe K., Fan X.C., Xia J. (2021). Photoacoustic Dual-Scan Mammoscope: Results from 38 Patients. Biomed. Opt. Express.

[83] Zheng E., Zhang H., Goswami S., Kabir I.E., Doyley M.M., Xia J. (2021). Second-Generation Dual Scan Mammoscope with Photoacoustic, Ultrasound, and Elastographic Imaging Capabilities. Front. Oncol..

[84] Butler R., Lavin P.T., Tucker F.L., Barke L.D., Böhm-Vélez M., Destounis S., Grobmyer S.R., Katzen J., Kist K.A., Makariou E.V. (2018). Optoacoustic Breast Imaging: Imaging-Pathology Correlation of Optoacoustic Features in Benign and Malignant Breast Masses. Am. J. Roentgenol..

[85] de Heer E.C., Jalving M., Harris A.L. (2020). HIFs, Angiogenesis, and Metabolism: Elusive Enemies in Breast Cancer. J. Clin. Investig..

[86] Li G., Huang Z., Tian H., Wu H., Zheng J., Wang M., Mo S., Chen Z., Xu J., Dong F. (2024). Deep Learning Combined with Attention Mechanisms to Assist Radiologists in Enhancing Breast Cancer Diagnosis: A Study on Photoacoustic Imaging. Biomed. Opt. Express.

[87] Fakhrejahani E., Torii M., Kitai T., Kanao S., Asao Y., Hashizume Y., Mikami Y., Yamaga I., Kataoka M., Sugie T. (2015). Clinical Report on the First Prototype of a Photoacoustic Tomography System with Dual Illumination for Breast Cancer Imaging. PLoS ONE.

[88] Zhang R., Zhao L., Zhao C., Wang M., Liu S., Li J., Zhao R., Wang R., Yang F., Zhu L. (2021). Exploring the Diagnostic Value of Photoacoustic Imaging for Breast Cancer: The Identification of Regional Photoacoustic Signal Differences of Breast Tumors. Biomed. Opt. Express.

[89] Zalev J., Richards L.M., Clingman B.A., Harris J., Cantu E., Menezes G.L., Avila C., Bertrand A., Saenz X., Miller S. (2019). Opto-Acoustic Imaging of Relative Blood Oxygen Saturation and Total Hemoglobin for Breast Cancer Diagnosis. J. Biomed. Opt..

[90] Tong X., Liu C.Z., Luo Y., Lin L., Dzubnar J., Invernizzi M., Delos Santos S., Zhang Y., Cao R., Hu P. (2025). Panoramic Photoacoustic Computed Tomography with Learning-Based Classification Enhances Breast Lesion Characterization. Nat. Biomed. Eng..

[91] Menezes G.L., Pijnappel R.M., Meeuwis C., Bisschops R., Veltman J., Lavin P.T., Van De Vijver M.J., Mann R.M. (2018). Downgrading of Breast Masses Suspicious for Cancer by Using Optoacoustic Breast Imaging. Radiology.

[92] Yang M., Zhao L., Yang F., Wang M., Su N., Zhao C., Gui Y., Wei Y., Zhang R., Li J. (2020). Quantitative Analysis of Breast Tumours Aided by Three-Dimensional Photoacoustic/Ultrasound Functional Imaging. Sci. Rep..

[93] Ge C., Gu I.Y.-H., Jakola A.S., Yang J. (2020). Enlarged Training Dataset by Pairwise GANs for Molecular-Based Brain Tumor Classification. IEEE Access.

[94] Zhan B., Xiao J., Cao C., Peng X., Zu C., Zhou J., Wang Y. (2022). Multi-Constraint Generative Adversarial Network for Dose Prediction in Radiotherapy. Med. Image Anal..

[95] Wolterink J.M., Leiner T., Viergever M.A., Išgum I. (2017). Generative Adversarial Networks for Noise Reduction in Low-Dose CT. IEEE Trans. Med. Imaging.

[96] Ko Y., Moon S., Baek J., Shim H. (2021). Rigid and Non-Rigid Motion Artifact Reduction in X-Ray CT Using Attention Module. Med. Image Anal..

[97] Yang G., Yu S., Dong H., Slabaugh G., Dragotti P.L., Ye X., Liu F., Arridge S., Keegan J., Guo Y. (2017). DAGAN: Deep de-Aliasing Generative Adversarial Networks for Fast Compressed Sensing MRI Reconstruction. IEEE Trans. Med. Imaging.

[98] Jiang J., Hu Y.-C., Tyagi N., Zhang P., Rimner A., Mageras G.S., Deasy J.O., Veeraraghavan H. (2018). Tumor-Aware, Adversarial Domain Adaptation from CT to MRI for Lung Cancer Segmentation.

[99] Tuysuzoglu A., Tan J., Eissa K., Kiraly A.P., Diallo M., Kamen A. (2018). Deep Adversarial Context-Aware Landmark Detection for Ultrasound Imaging.

[100] Ren J., Hacihaliloglu I., Singer E.A., Foran D.J., Qi X. (2018). Adversarial Domain Adaptation for Classification of Prostate Histopathology Whole-Slide Images.

[101] Zhang H., Goodfellow I., Metaxas D., Odena A. (2019). Self-Attention Generative Adversarial Networks.

[102] Vaswani A., Shazeer N., Parmar N., Uszkoreit J., Jones L., Gomez A.N., Kaiser Ł., Polosukhin I. (2017). Attention Is All You Need. Adv. Neural Inf. Process. Syst..

[103] Jiang Y., Chang S., Wang Z. (2021). Transgan: Two Pure Transformers Can Make One Strong Gan, and That Can Scale Up. Adv. Neural Inf. Process. Syst..

[104] Luo Y., Wang Y., Zu C., Zhan B., Wu X., Zhou J., Shen D., Zhou L. (2021). 3D Transformer-GAN for High-Quality PET Reconstruction.

[105] Ma Y., Yang C., Zhang J., Wang Y., Gao F., Gao F. Human Breast Numerical Model Generation Based on Deep Learning for Photoacoustic Imaging. Proceedings of the 2020 42nd Annual International Conference of the IEEE Engineering in Medicine & Biology Society (EMBC).

[106] Lou Y., Zhou W., Matthews T.P., Appleton C.M., Anastasio M.A. (2017). Generation of Anatomically Realistic Numerical Phantoms for Photoacoustic and Ultrasonic Breast Imaging. J. Biomed. Opt..

[107] John M., Barhumi I. (2025). Unrolled Deep Learning for Breast Cancer Detection Using Limited-View Photoacoustic Tomography Data. Med. Biol. Eng. Comput..

[108] Yamaga I., Kawaguchi-Sakita N., Asao Y., Matsumoto Y., Yoshikawa A., Fukui T., Takada M., Kataoka M., Kawashima M., Fakhrejahani E. (2018). Vascular Branching Point Counts Using Photoacoustic Imaging in the Superficial Layer of the Breast: A Potential Biomarker for Breast Cancer. Photoacoustics.

[109] Cocconi G., Di Blasio B., Alberti G., Bisagni G., Botti E., Peracchia G. (1984). Problems in Evaluating Response of Primary Breast Cancer to Systemic Therapy. Breast Cancer Res. Treat..

[110] Lin L., Tong X., Hu P., Invernizzi M., Lai L., Wang L.V. (2021). Photoacoustic Computed Tomography of Breast Cancer in Response to Neoadjuvant Chemotherapy. Adv. Sci..

[111] Gupta M.K., Qin R.-Y. (2003). Mechanism and Its Regulation of Tumor-Induced Angiogenesis. World J. Gastroenterol. WJG.

[112] Hohenberger P., Felgner C., Haensch W., Schlag P.M. (1998). Tumor Oxygenation Correlates with Molecular Growth Determinants in Breast Cancer. Breast Cancer Res. Treat..

[113] Garcia-Uribe A., Erpelding T.N., Krumholz A., Ke H., Maslov K., Appleton C., Margenthaler J.A., Wang L.V. (2015). Dual-Modality Photoacoustic and Ultrasound Imaging System for Noninvasive Sentinel Lymph Node Detection in Patients with Breast Cancer. Sci. Rep..

[114] Thomas V., BISDAS S. (2007). Lymph Node Staging. Top Magn Reson Imaging.

[115] Hsueh E.C., Hansen N., Giuliano A.E. (2000). Intraoperative Lymphatic Mapping and Sentinel Lymph Node Dissection in Breast Cancer. CA A Cancer J. Clin..

[116] Wang H., Liu S., Wang T., Zhang C., Feng T., Tian C. (2019). Three-dimensional Interventional Photoacoustic Imaging for Biopsy Needle Guidance with a Linear Array Transducer. J. Biophotonics.

[117] Piras D., Grijsen C., Schütte P., Steenbergen W., Manohar S. (2013). Photoacoustic Needle: Minimally Invasive Guidance to Biopsy. J. Biomed. Opt..

[118] Balasundaram G., Krafft C., Zhang R., Dev K., Bi R., Moothanchery M., Popp J., Olivo M. (2021). Biophotonic Technologies for Assessment of Breast Tumor Surgical Margins—A Review. J. Biophotonics.

[119] Imai T., Shi J., Wong T.T., Li L., Zhu L., Wang L.V. (2018). High-Throughput Ultraviolet Photoacoustic Microscopy with Multifocal Excitation. J. Biomed. Opt..

[120] Kim G.R., Kang J., Kwak J.Y., Chang J.H., Kim S.I., Youk J.H., Moon H.J., Kim M.J., Kim E.-K. (2014). Photoacoustic Imaging of Breast Microcalcifications: A Preliminary Study with 8-Gauge Core-Biopsied Breast Specimens. PLoS ONE.

[121] Grootendorst D.J., Jose J., Fratila R.M., Visscher M., Velders A.H., Ten Haken B., Van Leeuwen T.G., Steenbergen W., Manohar S., Ruers T.J. (2013). Evaluation of Superparamagnetic Iron Oxide Nanoparticles (Endorem^®^) as a Photoacoustic Contrast Agent for Intra-operative Nodal Staging. Contrast Media Mol. Imaging.

[122] Xi L., Zhou G., Gao N., Yang L., Gonzalo D.A., Hughes S.J., Jiang H. (2014). Photoacoustic and Fluorescence Image-Guided Surgery Using a Multifunctional Targeted Nanoprobe. Ann. Surg. Oncol..

[123] Chopra A. (2004). Molecular Imaging and Contrast Agent Database (MICAD).

[124] Gargiulo S., Albanese S., Mancini M. (2019). State-of-the-Art Preclinical Photoacoustic Imaging in Oncology: Recent Advances in Cancer Theranostics. Contrast Media Mol. Imaging.

[125] Zhang Y., Yu J., Kahkoska A.R., Gu Z. (2017). Photoacoustic Drug Delivery. Sensors.

[126] Moore C., Jokerst J.V. (2019). Strategies for Image-Guided Therapy, Surgery, and Drug Delivery Using Photoacoustic Imaging. Theranostics.

[127] Manivasagan P., Bharathiraja S., Bui N.Q., Jang B., Oh Y.-O., Lim I.G., Oh J. (2016). Doxorubicin-Loaded Fucoidan Capped Gold Nanoparticles for Drug Delivery and Photoacoustic Imaging. Int. J. Biol. Macromol..

[128] Park J., Choi S., Knieling F., Clingman B., Bohndiek S., Wang L.V., Kim C. (2025). Clinical Translation of Photoacoustic Imaging. Nat. Rev. Bioeng..

[129] (2025). Defining the Clinical Niche for Photoacoustic Imaging. Nat. Rev. Bioeng..

[130] Bohndiek S., Brunker J., Gröhl J., Hacker L., Joseph J., Vogt W.C., Armanetti P., Assi H., Bamber J.C., Beard P.C. (2019). International Photoacoustic Standardisation Consortium (IPASC): Overview (Conference Presentation).

[131] Bohndiek S. (2019). Addressing Photoacoustics Standards. Nat. Photonics.

